# Liver-draining portal lymph node responds to enteric nematode infection by generating highly parasite-specific follicular T helper and B cell responses

**DOI:** 10.3389/fimmu.2025.1483274

**Published:** 2025-02-28

**Authors:** Joshua Adjah, Zaneta D. Musimbi, Robert M. Mugo, Ankur Midha, Susanne Hartmann, Sebastian Rausch

**Affiliations:** Department of Veterinary Medicine, Institute of Immunology, Freie Universität Berlin, Berlin, Germany

**Keywords:** liver lymph nodes, enteric nematode, *Heligmosomoides polygyrus bakeri*, Th2 cells, germinal center B cells, PLN, CLN

## Abstract

**Introduction:**

While research on the gut-liver axis in non-communicable liver diseases has expanded exponentially, few studies have investigated the liver-gut relationship in the context of gastrointestinal nematode infections. This study aimed to determine whether liver-draining lymph nodes (LLNs) contribute to the immune response against a strictly enteric nematode infection.

**Methods:**

We analyzed the cellular and functional immune responses in the portal (PLN) and celiac (CLN) liver-draining lymph nodes following infection with the small intestinal nematode *Heligmosomoides (polygyrus) bakeri (H. bakeri)*. The composition of dendritic cells and CD4+ T cell subsets in LLNs was compared to the mesenteric lymph nodes (MLN), the primary draining site of gut infections. Additionally, we examined Th2 effector cell expansion, plasmablast generation, and B cell activation across these lymphoid sites.

**Results:**

Both PLN and CLN exhibited increased cellularity at d14 post-infection. The immune profile in CLN closely resembled that of MLN, characterized by a robust expansion of GATA-3+ Th2 effector cells at days 6 and 14 post-infection. This was accompanied by an early plasmablast response, producing low-affinity IgG1 antibodies targeting immune-dominant excretory-secretory (ES) products. In contrast, PLN showed weaker Th2 responses and lower early plasma cell responses compared to MLN and CLN. However, PLN displayed strong follicular T helper (TFH) activity, with a B cell profile biased toward germinal center reactions. This led to high-affinity IgG1 antibodies specifically binding VAL-1 and ACE-1.

**Discussion:**

These findings demonstrate, for the first time, that liver-draining lymph nodes actively participate in the adaptive immune response to enteric nematode infections. While MLN and CLN function synergistically in generating early Th2 effector cells and rapid extrafollicular IgG1+ plasma cell responses, PLN specializes in TFH-driven germinal center reactions and affinity maturation.

## Introduction

Generally, lymph nodes (LNs) play critical roles in the development of immune responses during infection (1–3). The generation of tissue-specific immunity is enhanced as a result of the LNs which are strategically located throughout the body (4). This permits simultaneous antagonistic immune responses in different organs in a concerted manner, thus providing a more controlled immune response in different organs (5).

This niche-specific immunity is particularly pronounced in the gastrointestinal tract, in which different gut segments are drained by immunologically distinct LNs to meet the different needs of these gut segments (6, 7). Supporting the concept of LN ‘sharing’ and co-drainage, several studies showed that gut-associated LNs also drain the pancreas and liver to varying extents (8–10). Given the intricate gut-liver cross-talk (11, 12), there is a potential for mixed drainage between the gut and liver LNs which is poorly understood to date.

Two liver-draining lymph nodes (LLN) are present in mice, the celiac lymph node, CLN, and the portal lymph node, PLN (8, 13). These LLNs lie adjacent to each other, with the PLN more superficial and located to the right of the portal vein while the CLN is located slightly deeper in the peritoneal cavity (14). The human liver shares this anatomical arrangement where it also seems to be drained by two sets of LNs with similar localization as in mice (15, 16). Of note, the nomenclature of the two murine LLNs differed between studies, but one theme runs through these studies; the consistency in description of the co-draining and organogenesis of the CLN and PLN (8, 14). The CLN co-drains some parts of the small intestine and the peritoneal cavity whereas the PLN drains the liver to a greater extent (5, 14). Here, we follow the nomenclature of Brown and colleagues (5).

Using Evans blue dye, Mayer et al., revealed the involvement of these LLNs in gut-restricted infection to be dependent on the route (subserosal into the intestines or footpad injection of antigens) of infection (17), showing migration of dendritic cells (DCs) from the liver to the LLNs to prime and facilitate specific T cell responses. Interestingly, CLN and PLN appear to be independent liver-draining LNs, with different cellular compositions and modes of organogenesis (18). Hence, the two LN may act differently when comparing hepatic vs. enteric infections but also comparing the responses of CLN and PLN in the context of any given intestinal infection. While we are not aware of studies testing the latter, a study comparing the CLN and PLN responses to hepatic virus infection reported that they were predominantly involved in liver antiviral immune responses (13). Accordingly, lymphadenectomy of the PLN resulted in hepatitis B virus (HBV) persistence in immunocompetent mice (14). The CLN on the other hand was shown to be important in promoting liver-mediated adaptive immune tolerance via induction of regulatory T cells (Tregs) and scarcity of DCs (13).

We, therefore, asked whether and how the two lymphoid organs contributed to the immune response against intestinal nematode infections, taking advantage of the strictly enteric infection with *H. bakeri*, a natural parasite of mice. Similar to widespread and economically important gastrointestinal nematodes of cattle and small ruminants, this worm develops through a short histotrophic phase of about 8 days, spent by the growing larval stages in the small intestinal submucosa, before returning to the lumen of the upper small intestine where the adult worms mate and survive for up to 9 months. We also compared the responses in CLN and PLN to those in the mesenteric lymph nodes (MLN).

In line with earlier evidence for the CLN draining parts of the upper small intestine, it has also been shown that the CLN co-drain liver, duodenum, and peritoneum (5, 14), hence a mix of liver-derived soluble signals/DC with the signals and APC from small intestines. The PLN on the other hand was shown to more exclusively drain the liver to a greater extent (5, 14). Thus, the present work seeks to address whether the LLNs contribute to the instruction of classical Th2 cells in a strictly enteric gastrointestinal nematode infection and whether the co-drainage ongoing between the MLN and LLNs could impact the immune responses in these LLNs fostering Treg, Th2, or B cells specializations in them.

We explored two-time points of *H. bakeri* infection, days 6 (early phase) and 14 (peak phase), representing the phases of larval development in small intestinal tissue and the arrival of the mature larvae in the lumen of the intestine, respectively. We demonstrate a clear distinction between the two LLNs; CLN, like the MLN, are strong Th2 induction sites, while the PLN is biased towards TFH cells and, hence, generates high-quality B cell responses during the infection.

## Results

### The liver-draining lymph nodes contribute to T effector cell responses against *H. bakeri* infection

**Figure 1:**
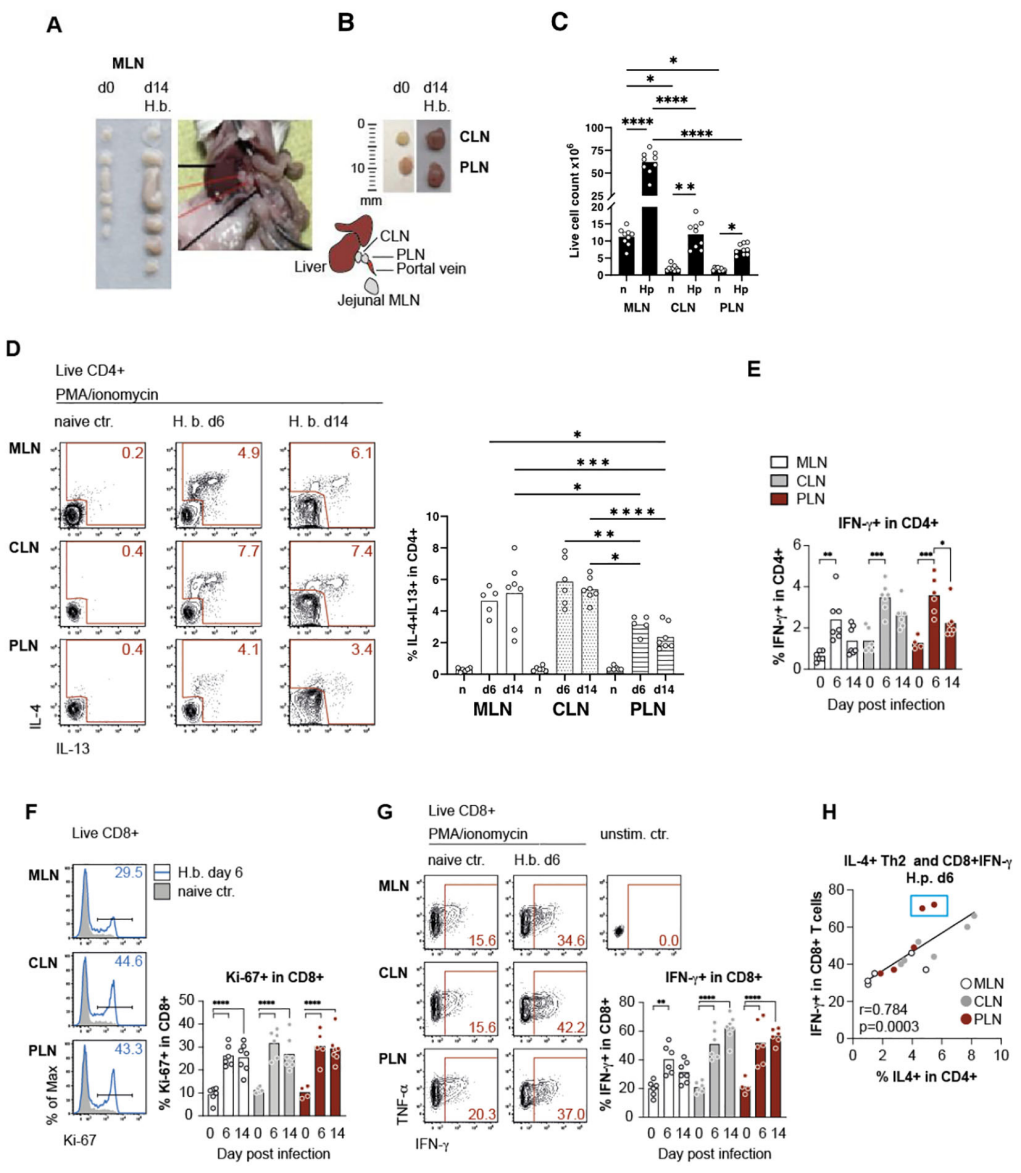
Mesenteric lymph nodes, in conjunction with liver-draining lymph nodes, contribute to T-cell effector responses against *H*. *bakeri* infection. **(A)** Mesenteric lymph nodes (MLN) at day 0 (d0) and day 14 (d14) post-infection with *H*. *b*, picture of BALB/c mouse dissected at day 14 to show the anatomical position of the two LLNs. **(B)** Image illustrating the jejunal MLN with the celiac (CLN) and portal lymph nodes (PLN), with a drawn scale for size comparison. Insets show the size of CLN and PLN on d0 and d14. **(C)** Quantification of live cell counts in MLN, CLN, and PLN on d0 and d14. Data are presented as mean ± SEM with statistical significance indicated. **(D)** Flow cytometry analysis of IL-4 and IL-13 expression in live CD4^+^ T cells (IL-4^+^ and IL-13^+^ double-positive cells gated within CD4^+^IL-4^+^ population) from MLN, CLN, and PLN on d0, d6, and d14 post-infection. Representative plots (left) and cumulative data (right) demonstrate an increase in IL-4^+^ and IL-13^+^ CD4^+^ T cells in infected mice. **(E)** Percentage of IFN-γ^+^ CD4^+^ T cells in MLN, CLN, and PLN on d0, d6, and d14 post-infection. Bars represent mean ± SEM, with statistical comparisons among time points. **(F)** Proliferation of live CD8^+^ T cells in MLN, CLN, and PLN on d6 post-infection with *H*. *b.* Representative histograms show Ki-67 expression in CD8^+^ T cells from naïve and infected mice (left). Bar graph (right) quantifying the percentage of Ki-67^+^ CD8^+^ T cells at different time points (d0, d6, and d14). **(G)** Cytokine production in live CD8^+^ T cells from MLN, CLN, and PLN at d6 and d14 post-infection. Representative flow cytometry plots show IFN-γ and TNF-α expression (left), while the bar graph quantifies the percentage of IFN-γ^+^ CD8^+^ T cells at each time point (right). **(H)** Correlation between IL-4^+^ Th2 cells and IFN-γ^+^ CD8^+^ T cells in MLN, CLN, and PLN at d6 post-infection. Each dot represents an individual mouse, with Pearson’s correlation coefficient (r) and p-value shown. Statistical significance: *p < 0.05, **p < 0.01, ***p < 0.001, ****p < 0.0001.

*H. bakeri* infects the small intestine of mice where the ingested infective larvae (L3) invade the submucosa of the duodenum and upper jejunum for development into the pre-adult stage, which returns to the small intestinal lumen at day 8 post-infection (19). The tissue damage provoked during the development of the histotropic larval stage results in a strong immune response reflected by the substantial increase in size and cellularity of the mesenteric lymph nodes (MLN) draining the duodenum and jejunum (**Figures 1A, B**). We further noticed that the size of the two lymph nodes draining the liver, namely celiac (CLN) and portal lymph nodes (PLN), were increased in mice infected with *H. bakeri* for 2 weeks (**Figures 1A–C**). Earlier studies demonstrated the multi-organ drainage by the CLN, comprising the liver and pancreas, but also the upper duodenum (5, 8, 13, 14), whereas the PLN solely drains the liver and pancreas (5). In accordance with the drainage of the upper SI shared between MLN and CLN, both sites comprised significantly elevated and similar frequencies of IL-4 and IL-13 competent Th2 cells during larval development (day 6 p.i.) and after transition to the patent stage of infection (day 14 p.i., **Figure 1D**). However, Th2 cells were also significantly increased in the PLN of infected mice at both timepoints, albeit at lower levels compared to MLN and CLN (**Figure 1D**). In parallel to the induction of Th2 cells, elevated IFN-g production by CD4+ T cells was seen in all LN samples at day 6, but the percentage of T-bet+ Th1 cells remained unchanged, and the rate of IFN-g production leveled down in CD4+ T cells at day 14 post-infection (**Figure 1E**).

We further sought to compare the expression of Th2 effector functions in the different lymph nodes and therefore assessed the activity of CD8+ T cells, as earlier work reported the IL-4 dependent expansion of CD8+ memory-like T cells which was increased along the expansion of CD4+ Th2 cells in *H. bakeri*-infected mice (20, 21). Confirming these earlier studies, we determined a highly significant increase in the proliferation of CD8+ T cells in all active lymph nodes of infected mice (**Figure 1F**). Infection-derived CD8+ T cells also displayed elevated T-bet expression, which translated to significantly stronger IFN-g responses upon *in vitro* stimulation compared to naïve controls (**Figure 1G** and data not shown). Interestingly, the comparable profiles observed across different lymph nodes suggest that Th2 cells are not only present but also actively induced in the PLN, where they contribute to the expansion of CD8+ VM cells as early as day 6 post-infection. This finding makes it unlikely that Th2 cells were initially induced in MLN or CLN and subsequently migrated to the PLN, as the PLN lacks a direct connection to the gut, a key site for Th2 induction.

Despite our claims that Th2 cell levels in the PLN are reduced by half compared to MLN and CLN (**Figure 1D**), the PLN exhibits a comparable magnitude of CD8+ T cell responses. This observation suggests that lower Th2 levels in the PLN, if present, do not negatively impact CD8+ T cell responses at day 6. Furthermore, IFN-g expression of CD8+ T cells was positively correlated with the extent of Th2 responses across the LNs.

Hence, while the majority of Th2 cells induced in response to a strictly enteric nematode infection derive from the larger MLN and CLN draining the site of infection, a more limited number of Th2 cells derive from the liver-draining PLN. Furthermore, the correlation between Th2 response and local expansion of memory-like CD8+ T cells indicates robust Th2 effector functions expressed in the active LNs. This included the liver-draining PLN which is activated during enteric nematode infection despite the lack of the lymphatics directly connecting the PLN to the site of infection.

### CLN and PLN display different gut-homing characteristics of GATA-3+ T cells and ALDH enzyme activity

As we observed that the LLNs are a site of Th2 induction during *H. bakeri* infection, we next sought to determine if these T effector cells (Teff) contribute to anti-helminth immunity in the gut. Therefore, we assessed the expression of the gut-homing marker CCR9 (22) in Th2 cells in the different lymph nodes. CCR9-mediated gut homing of GATA-3+ cells is shown to correlate with high expression of aldehyde dehydrogenase (ALDH) in DCs isolated from the MLN of mice early during infection (22, 23). In addition, the early stage of infection drives the early recruitment of T cells to the infected gut and determines worm clearance (24). We, therefore, looked at the gut-homing marker CCR9 at day 6 to get an idea of how these cells behave in extra-lymphoid compartments and their contributions to controlling gut infection.

**Figure 2:**
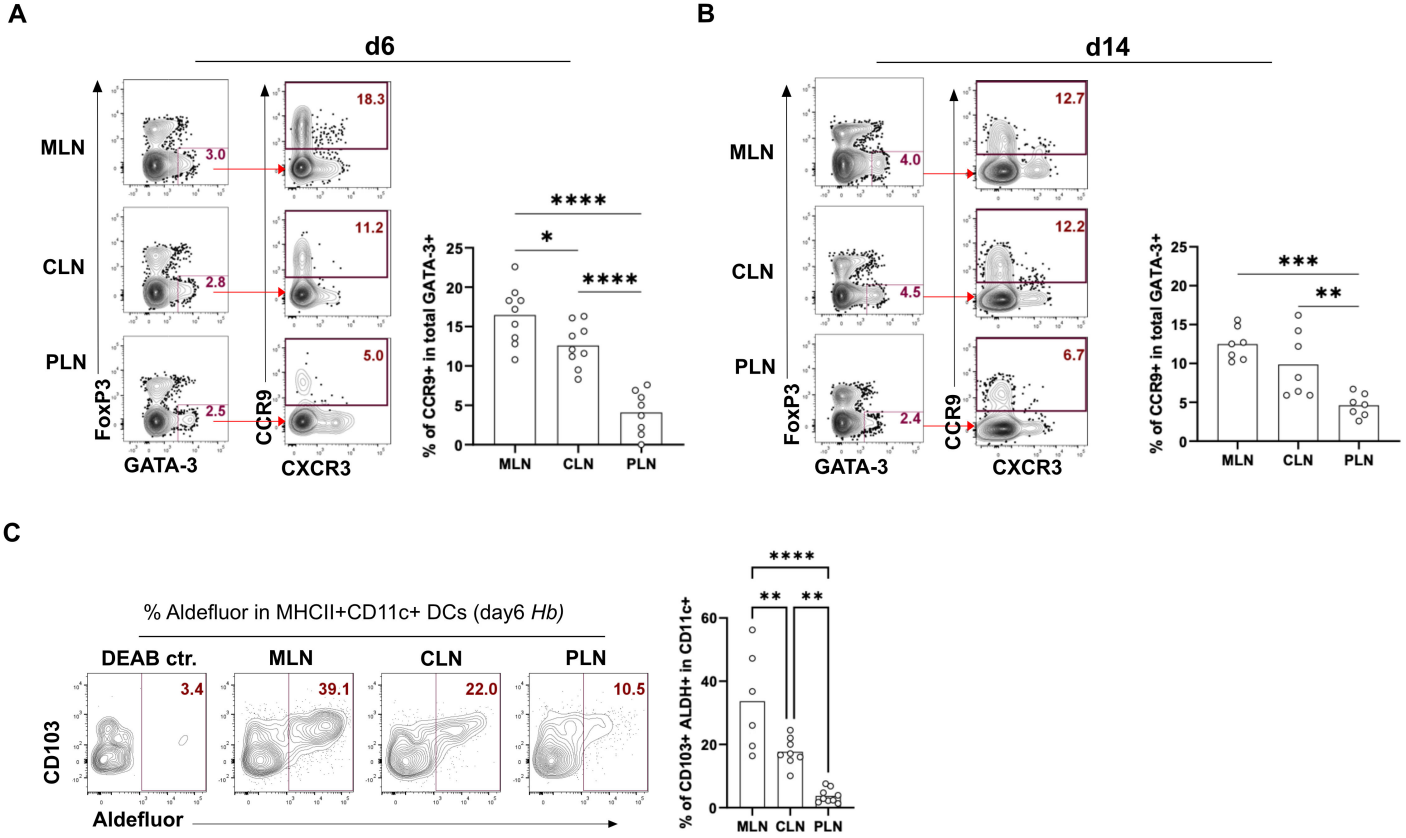
MLN, PLN, and CLN display differential expression of CD103+ ALDH+ DC along with differential gut homing of Th2. Frequencies of the gut homing marker CCR9 in total GATA-3+ Th2 cells in MLN, PLN, and CLN at **(A)** 6 days post-infection and **(B)** 14 days post-infection. Bar graphs report the mean percentages of CCR9+ in GATA-3+ Th2 (CD4+Foxp3- T helper cells) **(C)** Proportions of CD103+ ALDH+ DC in MLN, PLN, and CLN-derived cells on day 6 post-infection. Bar graphs report mean % of ALDH expression in MHCII+CD11c+CD103+ DCs. Cells were gated as CD8a- SiglecF- CD90- F4/80-. Cells were stained and analyzed by flow cytometry, representative FACS plots, and the data derived from at least two independent experiments performed with 3-4 mice per group shown. *p < 0.05, **p < 0.01, ***p < 0.001 and ****p < 0.0001 determined by Kruskal-Wallis test combined with Dunn’s multiple comparison test or Mann-Whitney test.

We observed that Th2 cells from the PLN express very low levels of CCR9 at days 6 and 14 compared to these cells in the CLN and MLN (**Figures 2A, B**). Higher RALDH activity (retinoic acid metabolism) in CD103+ MLN-DCs and an enhanced vitamin A metabolism during the early stage of infection resulted in higher CCR9+ T cells homing to the gut (25–27). Therefore, we assessed ALDH activity in CD103+ DCs at 6dpi using the Aldefluor kit. We found that low CCR9 expression in Th2 cells in the PLN was accompanied by low ALDH expression by CD103+ CD11c+ DCs. In contrast, the higher expression of gut-homing markers by Th2 cells from the CLN and MLN was mirrored by the higher expression of ALDH by DCs (**Figure 2C**). Together, this data shows that MLN and CLN express higher proportions of the gut-homing marker CCR9 compared to PLN as a result of higher levels of ALDH activity in their DCs.

### Differential B cell responses are associated with the magnitude of Th2 rather than TFH profiles

**Figure 3:**
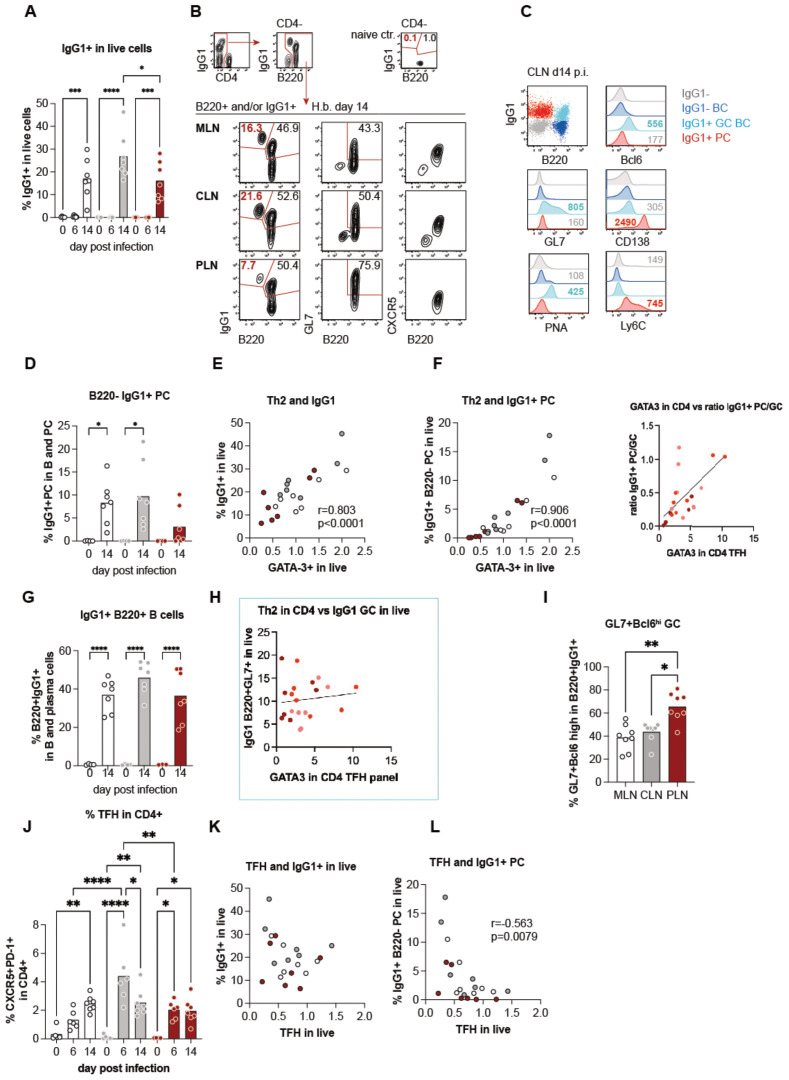
Differential B cell responses are associated with the magnitude of Th2 rather than TFH profiles. **(A)** Quantification of IgG1+ cells among live cells over the course of infection, showing a significant increase at day 14 post-infection. **(B)** Representative flow cytometry plots of B220+ and/or IgG1+ cells from mesenteric lymph nodes (MLN), cervical lymph nodes (CLN), and popliteal lymph nodes (PLN) at day 14 post-infection. Populations include germinal center(GC) B cells (B220+ GL7+ CXCR5+) and plasma cells (PCs; B220- IgG1+). **(C)** Flow cytometry analysis of IgG1+ subsets in CLN, highlighting markers associated with GC B cells (Bcl6, GL7) and PCs (CD138, Ly6C). **(D)** Percentage of B220- IgG1+ PCs across time points, showing significant expansion at day 14. **(E, F)** Correlation of Th2 marker GATA3+ in CD4+ T cells with IgG1+ cells **(E)** and B220- IgG1+ PCs **(F)** in live populations, with strong positive associations. **(G)** Proportions of B220+ IgG1+ GC B cells in B cell populations showing significant increases by day 14. **(H)** Correlation between GATA3 expression in CD4+ T follicular helper (TFH) cells and IgG1+ B220+ GC B cells, indicating a potential role of Th2 polarization. **(I)** Percentage of GL7+ Bcl6+ GC B cells within B220+ IgG1+ populations, showing a significantly higher proportion in the PLN compared to MLN and CLN. **(J)** Dynamics of CXCR5+ PD-1+ TFH cells in CD4+ T cell populations over time, peaking at day 14. **(K, L)** Negative correlation between TFH cell abundance and IgG1+ live cells **(K)** or B220- IgG1+ PCs **(L)**, suggesting TFH-independent regulation of PC responses.

The quantification of IL-4 and GATA-3 expression in CD4+ T cells in the liver versus gut-draining lymph nodes indicated a trend for less extensive Th2 effector cell generation in PLN compared to the ‘classical’ gut-draining lymph nodes. However, the three sites displayed similar features associated with IL-4 signaling in worm infections, such as the strong outgrowth of B cells and the expansion of VM CD8+ T cells (**Figure 1H**). Furthermore, both B cell proliferation and IFN-g competence of CD8+ T cells strongly correlated with the individual levels of Th2 responses in a given organ rather than with the organ itself (**Figure 1F**). We, therefore, asked if qualitative differences in the B cell responses might be associated with the extent of Th2 differentiation and/or the balance of Th2 effector versus follicular T helper cell differentiation across the different lymphatic sites. We hence quantified IgG1-expressing cells by intracellular stains at day 6 and 14 post-infection and found a significant rise of IgG1+ cells in the three lymph nodes at day 14 post-infection (**Figure 3A**). As shown for CLN-derived cells in **Figures 3B, C**, IgG1+ cells were composed of B220+ cells which displayed a germinal center (GC) B cell phenotype by the expression of the canonical GC transcription factor Bcl6, the binding of peanut agglutinin (PNA) and the elevated expression of GL7. In contrast, a smaller subset of B220-IgG1+ cells lacked GC markers and expressed CD138 and Ly6C associated with plasma cells (28). Furthermore, the B220- subset had lost the CXCR5 expression required for positioning in the B cell follicles (**Figures 3B, C**) (29–31). IgG1+ PC accounted for a prominent part of the IgG1+ cells in both MLN and CLN of infected compared to control mice (**Figure 3D**). In contrast, only 2 out of 8 mice harbored a prominent IgG1+ PC population in the liver-draining PLN at day 14 p.i, and these individuals also displayed the highest IgG1+ PC responses in MLN and CLN (**Figure 3D**). Because earlier studies demonstrated the capability of Th2 cells for the instruction of extrafollicular B cell responses (32, 33), we tested for associations between the magnitude of Th2 and overall IgG1 responses as well as early IgG1+ PC differentiation across the LN at day 14 p.i. As shown in **Figures 3E, F**, the frequencies of Th2 cells were strongly correlated with the overall outgrowth of IgG1+ cells (**Figure 3E**) as well as with the subset of IgG1+ B220- PC cells (**Figure 3F**). In contrast, the more homogenous GC B cell responses were not linked with the extent of Th2 differentiation (**Figures 3G, H**). Furthermore, while the PLN of most of the infected mice comprised low proportions of IgG1+ PC, the B220+IgG1+ subset was the most enriched in GL7+ Bcl6^high^ GC B cells in the liver-draining LN (**Figure 3I**, **Supplementary Figure 3**). These data suggested that the liver-draining PLN provided an environment suited for the generation of germinal center reactions while being less prone to the rapid instruction of plasma cell differentiation in the context of enteric nematode infection (**Supplementary Figure 3**).

We complemented the investigation of B and Th2 cell responses by the quantification of follicular T helper (TFH) cell response at days 6 and 14 post-infection. TFH cells are required for efficient germinal center reactions, resulting in the generation of high-affinity IgG and the development of memory B cells (34), whereas Th2 cells providing IL-4 were shown to be sufficient for extrafollicular IgG1 class switching and the generation of short-lived PC (35–37). As shown in **Figure 3J**, particularly high frequencies of TFH cells identified by the expression of the chemokine receptor CXCR5 together with the inhibitory signaling molecule PD-1 were detectable in CLN at day 6 post-infection. However, CD4 cells isolated from the three LN at day 14 post-infection comprised similar frequencies of TFH cells (**Figure 3J**, **Supplementary Figure 2**), matching the homogenous proportions of B220+IgG1+ GC B cells (**Figure 3G**). TFH responses were not mirrored in the overall rate of IgG1 class switching but negatively correlated with the extent of early plasma cell responses (**Figures 3K, L**).

### T cells in PLN are the only producers of parasite-specific IL-21 among the three LNs, driving efficient GC formation and high-quality antibody responses

IL‐21 has been reported to be integral to protective humoral immunity, where it acts on B cells from the outset of an adaptive immune response to promote their expansion and contribution to germinal center reaction (38–40). Therefore, to assess the contribution of IL-21 to the observed B cell responses, single-cell suspensions from the MLN, CLN, and PLN were stimulated with *H. bakeri* excretory-secretory product (HES) or antibodies against CD3 and CD28 (strong T cell receptor stimulants), cultured for 72 hours, after which the supernatants were harvested and tested for IgG1 and IL-21. We found that all three LNs harbor B cells producing IgG1 at day 14. However, we detected IgG1 in the supernatants of PLN cells on day 6 (**Figure 3A**), suggesting an early and sustained IgG1 production by B cells in this lymph node. Interestingly, of all the LNs, we could only detect IL-21 in the supernatant of cultured PLN cells in response to T-cell receptor stimulation (**Figures 3B, C**). In summary, these data suggest that the PLN environment is conducive in terms of IL-21 response by TFH and could support GC and quality B cell responses.

### B cells from PLN produce higher affinity IgG1 antibodies compared to MLN and CLN, which react to both VAL-1 and ACE-1

**Figure 4:**
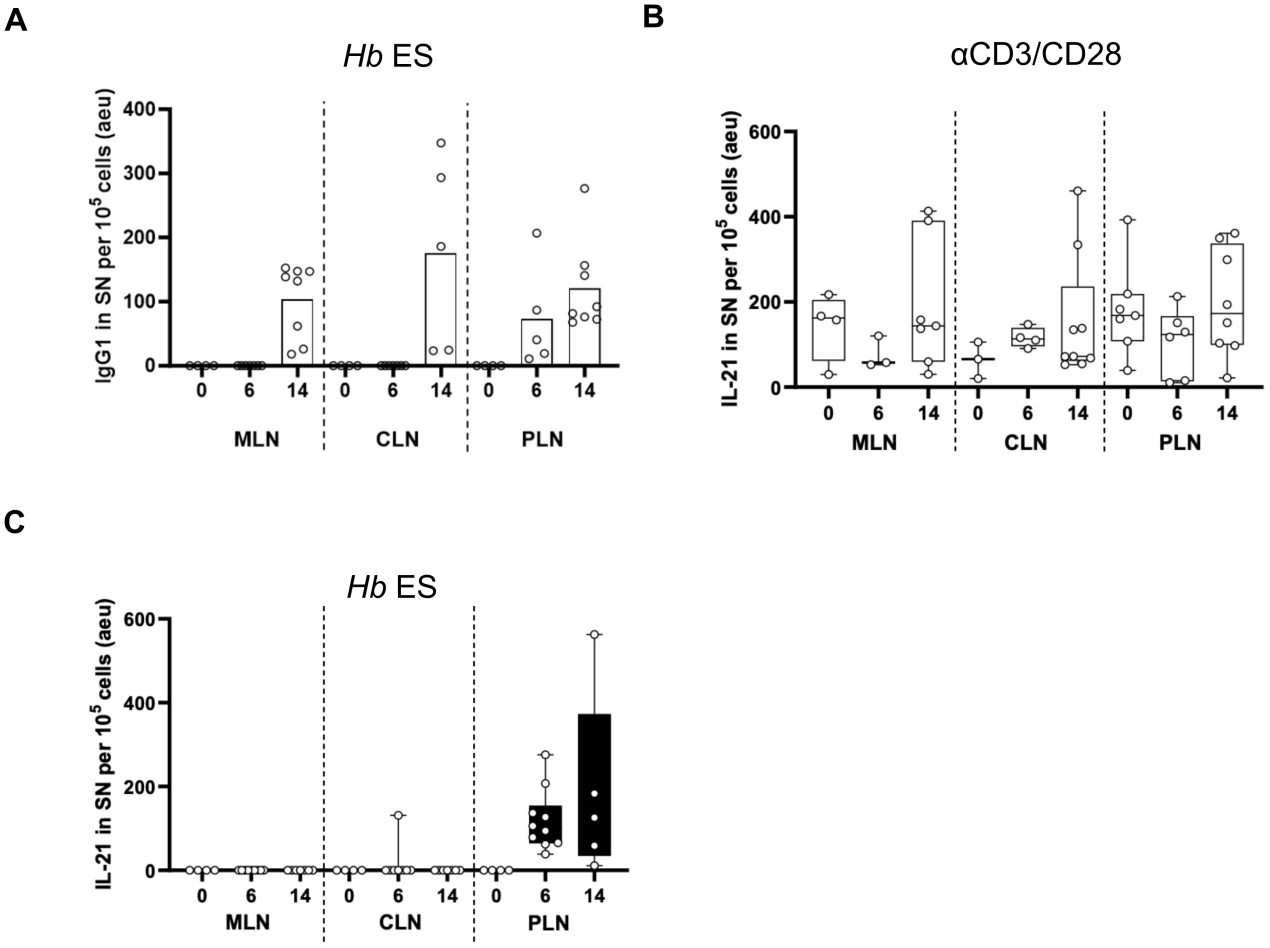
IgG1 and IL-21 profile in supernatants harvested after culturing cell suspensions of MLN, CLN, and PLN with *H. bakeri* excretory-secretory products (HES) **(A)** Arbitrary ELISA unit (EU) of IgG1 in the supernatant (SN) of cells cultured from MLN, PLN, and CLN at day 6 and 14 post-infection compared to steady state. **(B, C)** EU of IL-21 in supernatant (SN) of cells cultured from MLN, PLN, and CLN at day 6 and 14 post-infection compared to steady state. Data pooled from at least two independent experiments with 3-4 mice group is shown. Cells were stimulated with either anti-CD3/-CD28 **(B)** or *Hb* ES, *H*. *bakeri* excretory-secretory products **(C)**.

To confirm the assertion that the PLN environment supports higher quality B cell responses compared to the MLN and CLN, we run an SDS-PAGE of ‘untouched’ HES and de-glycosylated HES using either PNGase F or O-glycosidase. The HES was de-glycosylated to remove carbohydrate moieties (present in adult ES) on antigenic epitope, the so-called Glycan A and B, which have been shown to induce non-protective antibodies and act as a decoy that generates ineffective humoral responses during primary *H. bakeri* infection (41). From the SDS-PAGE gel, ‘untouched HES’ as well as de-glycosylated HES show the two band sizes that correspond to Venom allergen/Ancylostoma secreted protein-Like-1 (VAL-1) and acetylcholinesterase-1 (ACE-1) proteins, depicted by the red and black boxes, respectively (**Figure 4A**). These proteins are suggested to represent two major vaccine targets that induce protective immunity against *H. bakeri* (42). Western blots were thereafter run using pooled sera from d14 *H. bakeri* infected BALB/c mice as well as the supernatant of MLN, CLN, and PLN cell suspensions after 72 hours of culture with HES. We found that the serum IgG1 reacted strongly to these two bands (red and black boxes – **Figure 4B**).

Next, we probed whether the supernatants from MLN, CLN, and PLN show similar binding patterns. We show that the supernatants from the MLN and CLN only reacted to the second band, ACE-1 (black box) while the supernatant from the PLN reacted to the two bands just like the pooled sera (red and black boxes and red arrows – **Figure 4C**). We also show that both the ‘untouched’ and de-glycosylated HES showed similar binding patterns when probed with pooled sera or supernatant (**Figures 4B, C**). Furthermore, we show that with the supernatants, the binding to the de-glycosylated HES was weaker shown in fainter bands than the ‘untouched’ HES. The binding intensities to the ‘untouched’ HES decreased in MLN, compared to CLN and PLN, with the PLN showing the highest intensity. The data together show that the PLN has a similar binding pattern to the pooled sera, reacting to the *H. bakeri* vaccine target proteins VAL-1 and ACE-1.

In summary, our data show that the PLN, even though it has restricted Th2 responses, compared to the MLN and CLN, possibly due to its high IFN-γ environment, is skewed towards TFH response. Even though the PLN shows lower PCs compared to the MLN and CLN at day 14, the PLN is able to form more GC, which enables it to produce more quality B cells and high-affinity antibody response (**Figures 3D, I**), evident in the binding of its supernatants to both VAL-1 and ACE-1 compared to the MLN and CLN (**Figures 4A–C**). Together, our data shows that the PLN is important for quality B cell responses during *H. bakeri* infection.

## Discussion

Few studies of LNs in mice have identified cross-talks between gut-draining LNs and LNs at different anatomical locations (5, 8, 14). In mice, the LLNs have attracted attention only recently in the context of liver infections or gastrointestinal virus infections (5, 14). Not much work has been done on these LLNs in the context of intestinal helminth infection, especially considering the gut-liver axis and antigen sharing between these organs (43, 44). In previous studies using nematode models, many Th2 cells have been shown to circulate to the liver, but the phenotype and impact on the infection were not addressed (45–47). In our model, we observed increased LLN size even though *H. bakeri* does not traverse the liver. Thus, we speculated that there could be induction of Th2 in the LLNs that may support the MLN during infection. Furthermore, our data suggest that there is indeed a dissemination of parasite antigens to the liver, evidenced by significant levels of parasite-specific CD40L+ Th2 cells in the liver (unpublished data), which could be the result of type 2 effector responses induced by the two LLN.

We also showed that the two LLNs behaved differently in terms of gut-homing marker expression on their Th2 cells. Cells from the CLN typically had similar phenotypes to those from the MLN whilst the PLN cells differed. We show a higher number of Th2 cells co-expressing the gut homing marker, CCR9 in the MLN and CLN (**Figures 2A, B**) compared to the PLN. This is likely due to different properties of migratory DC sharing between lymph nodes, especially lymph nodes draining the gut and related to the gut. Migratory DC sharing is important in fostering cross-talk and the imprinting of certain phenotypes (like gut-homing markers) on T cells in the different lymph nodes involved (5). The PLN is probably paramount in contributing to the high systemic Th2/1 hybrid cells, possibly due to their poor CCR9 expression (**Figures 2A, B**) (48). This we also confirmed by the ALDH levels and activity of CD103+ DC (**Figure 2C**), showing significantly fewer levels of these cells in the PLN compared to the CLN and MLN.

PLN has been shown to share migratory DC with the liver during a hepatotropic viral infection to a greater extent than CLN (5, 8, 13). This suggests an active role of the PLN in liver-specific infections. This phenomenon has also been reported by Brown and colleagues, who show that migratory DCs are shared to a greater extent between CLN and MLN than PLN (5). Alternatively, more antigen-primed migratory DC have access to the CLN compared to the PLN, perhaps due to its proximity to the intestine. The PLN might not be involved in tolerance but rather effector mechanisms and is probably the more important lymph node (in terms of draining the liver) for the liver during *H. bakeri* infection. This could also imply an extra role for the PLN’s involvement in the mounting immune response to hepatic infections (8) aside from its somewhat involvement in intestinal infection. The PLN is probably involved in later T cell response or dealing with the excesses of the infection; for instance, gut translocated microbes (as a result of gut barrier dysfunction during the worm’s life cycle) (49). We speculate this is due to the high IFN-γ competence of the Th2 PLN cells (Th2/1 hybrid cells) (50) compared to the other LNs (**Supplementary Figure 1**). This would have to be further investigated.

Our work and the work of others defined the MLN as the main site of Th2 generation and IgG1 class switching in *H. p. bakeri* infection (51–53). Here, we show that the two LLNs contribute to Th2 and TFH generation and the instruction of IgG1 responses. In accordance with the drainage of duodenal tissue by the CLN (5), the reaction of this LN largely phenocopied the MLN response according to the parameters investigated in this study. Despite potential additional cellular and soluble messenger input from the liver and peritoneal cavity, the CLN harbored about the same frequencies of GATA-3 high Th2 cell marked by the co-production of IL-4/-13 and a similar fraction of IL-4, -13, and -5 triple-competent cells as seen in MLN. In addition, both MLN and CLN displayed similar proportions of CXCR5+PD1+ TFH cells and comparable B cells IgG1 class switching (**Figure 3J**). As anticipated by the increase in size, also the PLN responded to the enteric infection. The frequencies of Th2 cells were only reduced by about 50% compared to MLN and CLN, which is remarkable as the PLN, to the best of our knowledge, lacks any afferent lymphatic connection to the intestine.

More striking, the TFH response expressed in PLN upon 6-14 days of *H. bakeri* infection mirrored those generated in MLN and CLN and the extent of IgG class switching was alike in the B cell populations of all three LNs. These responses were apparently generated with similar kinetics, as Th2 cells almost exclusively expressing high levels of Ki67 were prominent and in all investigated LN at d6 post-infection (**Figure 1F**). These data suggest that the strong Th2 responses dominating the MLN and CLN might interfere with optimal TFH and B cell interactions (54). However, the stratification of Th2 (IL4, GATA3 expression), TFH, and IgG1(EF/GC) and the associated gradual differences in Th2 effector cell counts did not reveal a dependence on EF/GC phenotypical composition of B cells. These data are, however, limited to MLN and spleen (not shown here), and similar investigations may result in different outcomes concerning the responses in CLN/PLN.

**Figure 5:**
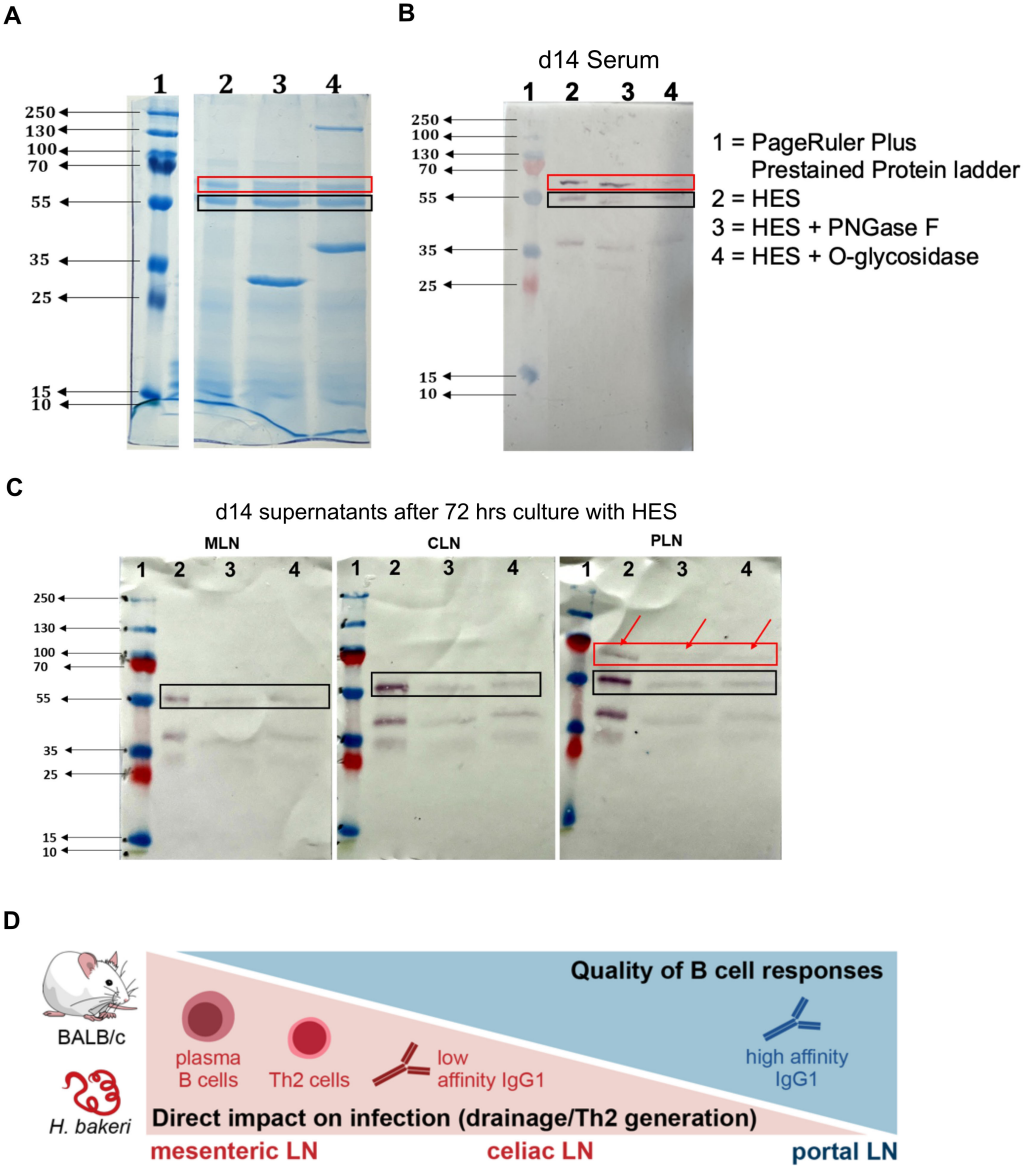
B cells from PLN produce higher affinity IgG1 antibodies compared to MLN and CLN, that react to both VAL-1 and ACE-1 of *H*. *bakeri* excretory-secretory product (HES) **(A)** SDS-PAGE of ‘untouched’ HES and de-glycosylated HES. Proteins were loaded at 10ug **(B)** Western blot of SDS-PAGE in **(A)** using pooled sera from day 14 of *H*. *bakeri* infected BALB/c mice **(C)** Western blot of SDS-PAGE in **(A)** using supernatants (1:2 dilution) from MLN, CLN and PLN cell suspensions cultures with HES for 72 hours from day 14 of *H*. *bakeri* infected BALB/c mice. Goat anti-mouse IgG1 conjugated to AP was used as a secondary antibody and BCIP/NBT as substrate. **(D)** A graphical summary of a suggested model for our findings. TFH and B cell response (and the lower Th2) are generated in PLN, which is not directly connected to the gut via lymphatics. But it may act as an overflow/2^nd^ line of response to the gut infection as a result of a few DCs moving from the MLN to the PLN. This might provide the optimal conditions for a more appropriate/specific antibody response compared to the less specific B cell responses in MLN and CLN.

During an ongoing infection, lower affinity B cells express IgM and IgD but can re-enter GC and form new PC that produces high-affinity antibodies (55). There are a lot more B cells re-entering or forming GC in the PLN compared to the MLN and CLN. This is evident in the GC formation ability in PLN compared to the MLN and CLN (**Figures 3I**, **5C**). IFN-γ signaling has also been reported to be very important in spontaneous GC formation (56). The PLN’s highly IFN-γ competent environment (from Th2 cells, **Supplementary Figure 1**) during the infection could be driving the high GC formation in the PLN. Also, its lower IL-4 competence compared to the other LNs during infection could foster its GC-forming ability (54). These suggest that, even though earlier on in the infection, you have good PCs from MLN and CLN, these PC may not be providing high-affinity antibodies that are necessary for the infection. However, the PLN, which initially formed lower PCs, will later have their B cells re-entering the GC, where they are ‘reprogrammed’ to produce higher affinity and more quality antibody responses (**Figures 5C, D**). IL‐21 production by TFH (57, 58) and calcium signaling via NFAT (59, 60) enable IL‐21 to fine‐tune B cell responses in relation to the immunological properties of the immunogen. This cytokine has also been shown to promote early B cell expansion by increasing cell cycle speed and cyclic re‐entry, synergizing with BCR and CD40 to increase AKT and S6 phosphorylation (60–62). The consequence for B cell responses is that initially, a wide range of antigen affinities are promoted, increasing B cell response size and promotion of plasma cells (63, 64). PLN showed a sustained IgG1 response during the early and peak stages of *H. bakeri* infection. It was also the only LN, in whose cell suspension IL-21 was detected after culturing for 72 hours. This further confirms our earlier thought that the PLN is more biased towards TFH, due to its lower Th2 response (**Supplementary Figure 2**), which makes it a good site for the generation of more quality B cell responses later in the infection.

Furthermore, protective immunity against *H. bakeri* infection has been achieved by vaccination with adult HES (41). Analyses of serum from vaccinated mice identified two major antigens of the venom allergen and acetylcholinesterase families recognized by the protective immune response as VAL-1 and ACE-1, respectively. These two antigens have been thought to be a major vaccine target for *H. bakeri* infection (42). Our data shows that supernatants from MLN and CLN of day 14 *H. bakeri*-infected BALB/c mice could only react to ACE-1 (black box – **Figure 5C**), suggesting the absence of B cell clones that produce IgG1 against VAL-1. This phenomenon could be suggestive of the early PC expansion in the MLN and CLN (**Figure 5A**), which leads to the production of low-affinity IgG1 antibodies that do not recognize epitopes of the VAL-1 proteins (**Figure 5C**). The PLN, on the other hand, had a lower PC population at day 14 but formed more GC (**Figure 3D**), leading to the B cells churning out high-affinity IgG1 antibodies that are fully functional and reactive to both VAL-1 and ACE-1 (**Figures 5B, C**). The pooled sera also reacting to both VAL-1 and ACE-1 suggests that the VAL-1-specific IgG1 in the serum derives from the PLN and not the MLN and CLN (**Figures 5B, C**). Putting these together, we report other lymphoid compartments that are important in the generation of B cell response during this infection. Our data suggest that the CLN and PLN are as important in mounting good B-cell responses just as the MLN. The PLN contributes to more sustained antibody responses by the formation of more GCs during the infection to enhance high-affinity antibody responses, which is necessary for protective immunity in *H. bakeri* infection.

In conclusion, GATA-3+ effector cells derive from the two LLNs in addition to the MLN during *H. bakeri* infection. The CLN behaves similarly to the MLN due to the fact that it also drains the gut and peritoneal cavity alongside the liver compared to the PLN which strictly drains the liver (5). The MLN and CLN are the more active LNs in generating T effector responses in *H. bakeri* infection. The PLN, on the other hand, is more important in generating quality B cell responses to enhance high-affinity antibody (IgG1) secretion, a consequence of it being a more TFH-skewed microenvironment. Further studies will be needed to demonstrate that the T and B cells induced in the LLNs are having a direct effect on *H. bakeri*, by either lymphodectomy or selective blocking of T and B cell egress from the LLNs. Also, the adoptive transfer of the cells from the LLNs could reveal other interesting findings of these LNs during *H. bakeri* infection.

## Materials and methods

### Mice and infections

The animal experiments were done in accordance with the National Animal Protection Guidelines with approval from the German Animal Ethics Committee for the Protection of Animals (G0176/17, G0113/15, H0438/17). Female BALB/c mice were ordered from Janvier (Saint-Berthevin, France) and were infected with 200 3rd-stage larvae of *H. bakeri* by oral gavage. Mice used in the experiments were anesthetized with xylazine and ketamine and sacrificed by cervical dislocation. All animal experiments were performed in accordance with the National Animal Protection Guidelines and approved by the German Animal Ethics Committee for the Protection of Animals (LAGeSo, G0176/20).

### Cell isolation

MLN, CLN, and PLN were processed into single-cell suspensions using 70 µl cell strainers (BD Biosciences, San Jose, CA, USA). After washing in RPMI (1% FCS, 100U/ml penicillin, 100 mg/ml streptomycin (PAA, Wien, Austria), any residue red blood cells were removed with an erythrocyte lysis buffer, followed by two further washing steps.

### Flow cytometry

For the detection of gut homing markers, cells isolated from lymphatic organs were stained for CCR9 (clone CW-1.2; PE-Cy7) and α4β7 (clone DATK32, biotin) at 37°C for 30 minutes, followed by incubation with a fixable viability dye (eFluor506 or eFluor780) on ice for 5 minutes. After labeling CD4 (clone RM4-5; Alexa 700, Brilliant Violet 510, or PerCP), cells were fixed using fix/perm buffer (Thermo Fisher) for 30 minutes at room temperature and stained intracellularly using the following reagents: FoxP3 (clone FJK-16s; PE-eFluor610, Alexa 488, or PerCP-Cy5.5), GATA-3 (clone TWAJ; eFluor 660), T-bet (clone 4B10; PE), and Ki-67 (SolA15, eFluor 450 or PE-Cy7). Streptavidin coupled with Brilliant Violet 605 was used as a secondary conjugate. Dendritic cells were analyzed using antibodies against MHCII (clone M5/114.15.2; APC), CD11c (clone N418; Brilliant Violet 421), and CD103 (clone 2E7, APC). Aldehyde dehydrogenase (ALDH) activity was determined using the Aldefluor kit (Stemcell Technologies). Non-specific binding of antibodies was prevented by adding FcγRII/III blocking antibody (clone 93). All antibodies and other reagents were from BioLegend, Thermo Fisher, or BD Biosciences. A catalog of the respective antibodies used in the multi-color flow cytometry can be seen in **Supplementary Table 1.1** in the **Supplementary Figures**. Cells were acquired on FACSCantoTM II (BD Biosciences) or FACSAriaTM III (BD Biosciences), and data was analyzed using FlowJo (Tree Star Inc., Ashland, USA). The antibodies used for the detection of surface and intracellular markers are described in **Supplementary Table S1**. Dead cells were excluded using eFluor780 or eF560 fixable viability dye (Thermo Fisher, Waltham, USA). For intracellular staining of cytokines and transcription factors, cells were fixed and permeabilized using the Fixation/Permeabilization kit and Permeabilization buffer from ThermoFisher/eBioscience. Analyses of the samples were done using a Canto II flow cytometer and an Aria cell sorter (BD Biosciences, Heidelberg, Germany) and FlowJo software Version 10 (Tree Star Inc., Ashland, OR, USA) was used to analyze the data. The data for naïve and infected mice (days 6 and 14) presented in this study are pooled from at least two independent experiments with 3-4 mice per group in each experiment.

### Cell culture *and in vitro* re-stimulation

For the analysis of cytokine production capacities of cells, MLN, CLN, and PLN cells were plated out per well in a round-bottom 96-well cell culture plate in a final volume of 200μL RPMI medium, containing 10% FCS, 100U/mL penicillin, and 100μg/mL streptomycin (all from PAA, Pasching, Austria). The cells were then stimulated with 1μg/mL of PMA/ionomycin and incubated for 3-4 hours at 37°C and 5% CO_2_. The cells were further stained with a dead cell marker and other surface stains, fixed and permeabilized for intracellular staining, followed by acquisition via flow cytometry.

### Parasite-specific IgG1 and IL-21 detection by ELISA

For the analysis of parasite-specific IgG1 and IL-21, 1x10^5^ of MLN, CLN, and PLN cells were plated out per well in a round-bottom 96-well cell culture plate in a final volume of 200μL RPMI medium containing 10% FCS, 100U/mL penicillin and 100μg/mL streptomycin (all from PAA, Pasching, Austria). The cells were stimulated with either anti-HES (10μg/mL) or without. The cells were then incubated for 72 hours at 37°C and 5% CO_2_. The cell suspensions were then spun down, and the supernatant was harvested and measured via sandwich. Absorbance was measured on a Biotek Synergy H1 Hybrid Reader at a 450nm wavelength.

### HES preparations, SDS-PAGE, and Western blots

Adult *H. bakeri* excretory and secretory product (HES) were either deglycosylated with Peptide N-glycosidase F (PNGase F), which cleaves N-linked glycans from glycoproteins, or Endo-α-N-Acetylgalactosaminidase (O-glycosidase), that catalyzes the removal of O-disaccharides from glycoproteins. These enzymes are from New England Biolabs^®^. Glycosylated and deglycosylated HES were run on an SDS-PAGE, and Western blot was done on these using either serum from d14 *H. bakeri* infected BALB/c mice or supernatants from cell suspension from MLN, CLN, and PLN after culturing with HES for 72 hours according to the protocol published in (65). Briefly, the SDS-PAGE was blotted unto a nitrocellulose membrane, blots were blocked in 2% dry skim milk in TBS with 0.05% Tween 20 for 2 hours at room temperature and probed with either pooled sera (1:200 dilutions) or supernatants from cell suspensions of MLN, CLN, and PLN (1:2 dilutions) at 4 ^0^C overnight. This was followed by 3 times washing in TBST, after which AP-conjugated secondary antibodies (anti-mouse IgG1 1:3000, antibodies-online.com) for 2 hours at room temperature. The blots were then washed 3 times in TBST and developed with SIGMAFAST™ BCIP^®^/NBT substrate (Sigma-Aldrich^®^).

### Statistics

All statistical analyses were performed using GraphPad Prism Software (San Diego, CA, USA). Normality was tested with the Shapiro-Wilk test, followed by ordinary one-way-ANOVA or Kruskal-Wallis test and Tukey’s or Dunn’s multiple comparison test. Comparisons of the two groups were performed with an unpaired t-test or Mann-Whitney test.

## Data Availability

The original contributions presented in the study are included in the article/**Supplementary Material**. Further inquiries can be directed to the corresponding author.
