# Supplementary material for: Liver-draining portal lymph node responds to enteric nematode infection by generating highly parasite-specific follicular T helper and B cell responses
Source: Front Immunol. 2025 Feb 28;16:1483274. doi: 10.3389/fimmu.2025.1483274 (PMC11906467; doi:10.3389/fimmu.2025.1483274)
Supplement: Supplementary file 1 [file DataSheet1.pdf]

# Supplementary Material

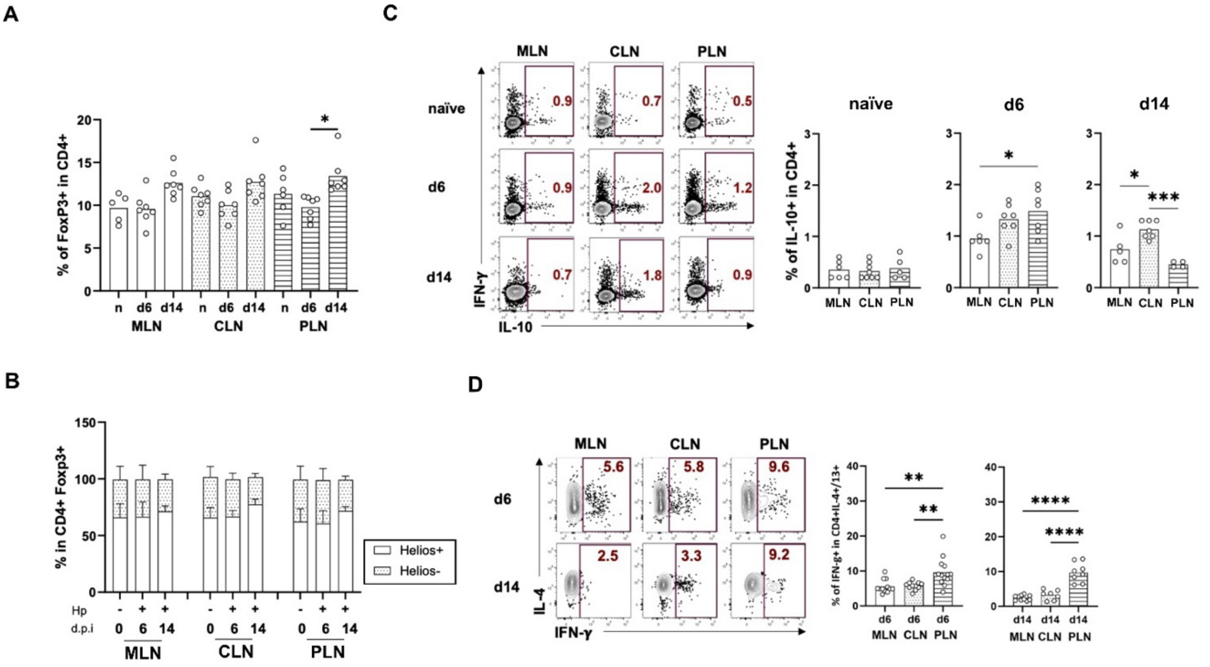


**Suppl. 1. Proportions of CD4+FoxP3+ Tregs and Helios in MLN, PLN, and CLN are similar, however, IL-10 production and *IFN-γ populations* differ at day 6 and 14 post-infection.** (**A**) Bar graphs showing the mean proportions of FoxP3+ in CD4+ T cells in MLN, PLN, and CLN at days 6 and 14 post-infection. (**B**) Staked bar graphs showing mean proportions and frequencies of Helios+/- cells in FoxP3+ CD4+ T cells in MLN, PLN, and CLN at 6 and 14 post-infections. (**C**) FACS plots showing frequencies and proportions of IL-10 in CD4+ IL-4+/IL-13+ cells in MLN, PLN, and CLN comparing naïve mice to day 6 and 14 post-infection. Bar plots showing the mean % of IL-10+ cells in CD4+IL-4/13+ T cell population comparing steady state and day 6 and 14 post-infection. (**D**) FACS plots showing frequencies and proportions of IFN-**γ** in CD4+IL-4+/IL-13+ cells in MLN, PLN, and CLN at days 6 and 14 post-infection. Bar plots showing the mean % of IFN-**γ** + cells in the CD4+ IL-4+/IL-13+ T cell population comparing days 6 and 14 post-infection. Data from at least two independent experiments with 3-4 mice per group is shown. * $p < 0.05$, ** $p < 0.01$, *** $p < 0.001$, **** $p < 0.0001$ determined by Kruskal-Wallis test combined with Dunn's multiple comparison test or Mann-Whitney test.

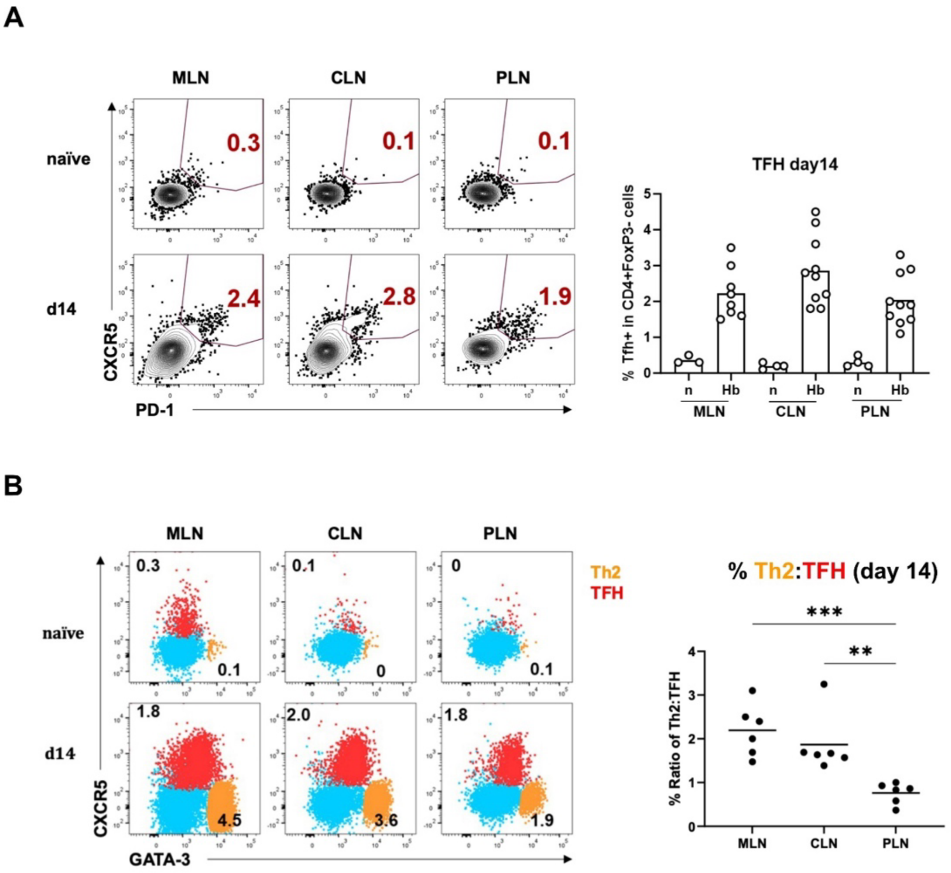


**Suppl. 2. TFH cell profiles of MLN, CLN, and PLN** (**A**) Proportions of CXCR5+PD-1+ TFH cells in the different lymphoid compartments at day 14 post primary infection. Bar graphs show the mean % of CXCR5+PD-1+ TFH cells in CD4+FoxP3- cells at day 14 post-infection in MLN, CLN, PLN, and spleen (**B**) Representative FACS plots show cell populations of Th2 cells (yellow population) and TFH cells (red population) at steady state and at day 14 post-*H. bakeri* infection. Corresponding dot plots showing the ratio of Th2 to TFH cells in the three lymph nodes (MLN, CLN, and PLN) translating into the bias of these lymph nodes to either Th2 or TFH response. Cells were stained and analyzed by flow cytometry, representative FACS plots, and the data derived from at least two independent experiments performed with 3-4 mice per group shown. ** $p < 0.01$, *** $p < 0.001$ determined by the Kruskal-Wallis test combined with Dunn's multiple comparison test or Mann-Whitney test.

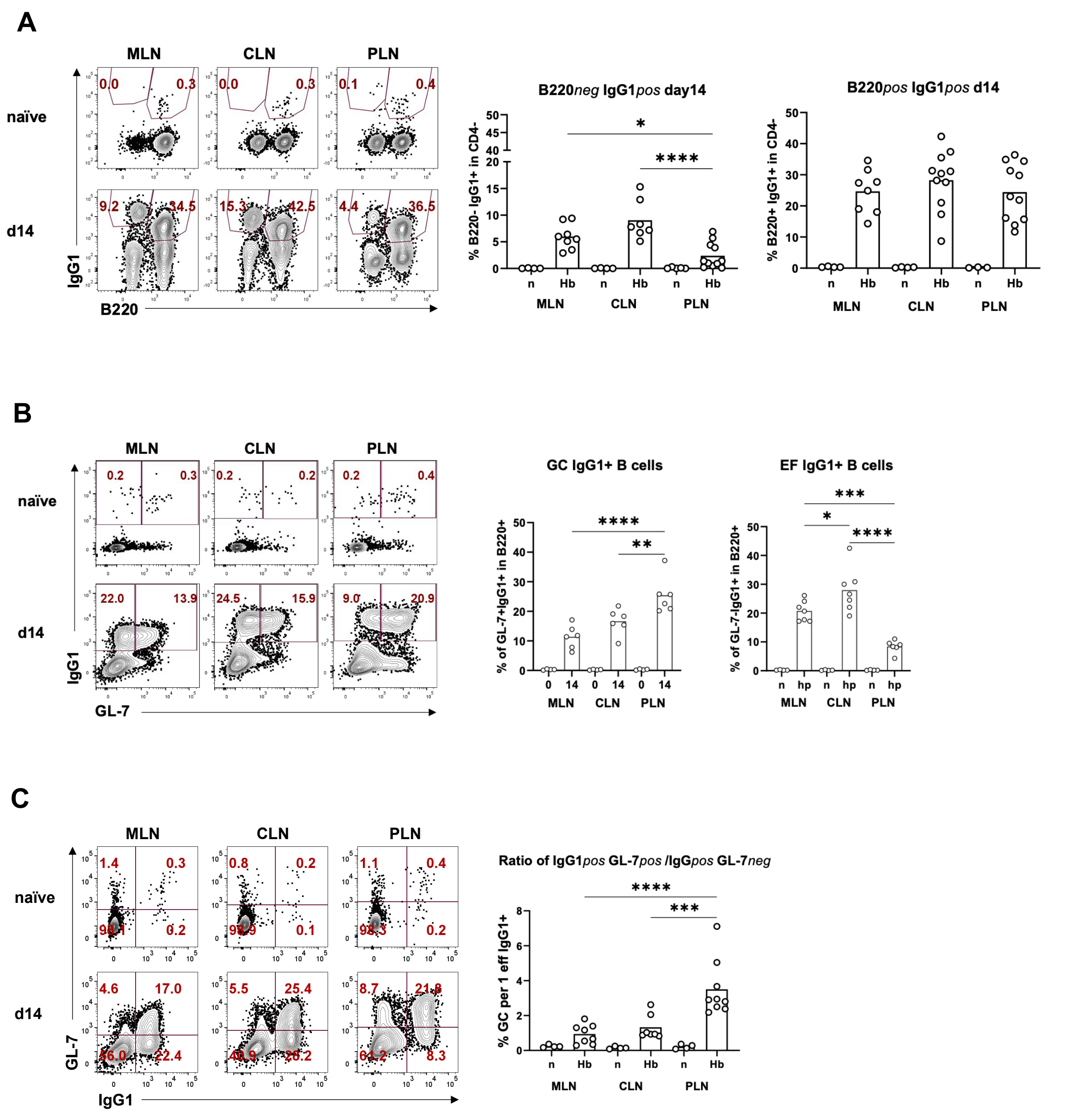


**Suppl. 3. MLN, CLN, and PLN display similar proportions of IgG1+B220+ B cells, however, PLN has a poor plasma B cell response compared to the other lymph nodes** (**A**) Proportions of IgG1+ (plasma B cells) and IgG1+B220+ (possibly becoming memory B cells) in MLN, CLN, and PLN at day 14 post-primary infection. Bar graphs showing the mean % of B220-IgG1+ (plasma cells) and B220+IgG1+ in CD4- cells at day 14 post primary infection. (**B**) Representative FACS plots showing the proportions of GL-7+IgG1+ germinal center (GC) B cells and GL-7-IgG1+ extrafollicular (EF) B cells in the different lymph nodes. Bar plots showing the mean % or proportions of GL-7+IgG1+ GC B cells GL-7-IgG1+ EF B cells and (**C**) Representative FACS plots and a corresponding bar graph showing the ratio of IgG1+GL-7+ GC B cell per one GL-7-IgG1+ extrafollicular B cell in the MLN, CLN, and PLN. Data shown from at least two independent experiments performed with 3-4 mice per group is shown. ** $p < 0.01$, *** $p < 0.001$, **** $p < 0.0001$ determined by the Kruskal-Wallis test combined with Dunn's multiple comparison test or the Mann-Whitney test.

**Suppl. 1 Table of antibodies for multi-colour flow cytometry**

| Marker | Colour | Company | Clone |
|---|---|---|---|
| **CCR9** | PE-Cy7 | Life Technologies /Thermo Fischer scientific | CW-1.2 |
| **α4β7** | Biotin | Life Technologies /Thermo Fischer scientific | DATK32 |
| **Streptavidin** | Qdot | BioLegend | |
| **CD45** | A700 | BioLegend | 30-F11 |
| **CD103** | A647 | Life Technologies /Thermo Fischer scientific | 2E7 |
| **MHCII** | APC | Life Technologies /Thermo Fischer scientific | M5/114.15.2 |
| **Aldefluor** | FITC | Stemcell Technologies | |
| **GATA-3** | APC | Life Technologies /Thermo Fischer scientific | TWAJ |
| **T-bet** | PE | Life Technologies /Thermo Fischer scientific | 4B10 |
| **CXCR3** | A405 | BioLegend | CXCR3-173 |
| **Helios** | FITC | Life Technologies /Thermo Fischer scientific | 22F6 |
| **FoxP3** | Texas-Red | Life Technologies /Thermo Fischer scientific | FJK-16s |
| **B220** | A700 | BioLegend | RA3-6B2 |
| **PD-1** | APC-Cy7 | Life Technologies /Thermo Fischer scientific | J43 |
| **Stretavidin** | PE-Cy7 | Life Technologies /Thermo Fischer scientific | |
| **IgG1** | BV421 | BioLegend | RMG1-1 |
| **CXCR5** | Biotin | BD Bioscience | |
| **Bcl-6** | PE | Life Technologies /Thermo Fischer scientific | BCL-DWN |
| **IL-10** | APC | BD Bioscience | JES5-16E3 |
| **IL-4** | PE-Cy7 | Life Technologies /Thermo Fischer scientific | 11B11 |
| **IL-13** | PE-Cy5 | Life Technologies /Thermo Fischer scientific | eBio13A |
| **CD4** | A700/A430/Qdot 605 | BD Bioscience/ Life Technologies /Thermo Fischer scientific /BD Bioscience | RM4-5 |
| **CD11b** | APC-Cy7 | BD Bioscience | M1/70 |

| | | | |
|---|---|---|---|
| **CD11c** | A450 | Life Technologies /Thermo Fischer scientific | N418 |
| **CD90** | PE-Cy5 | BioLegend | 30-H12 |
| **Siglec-F** | BV421 | BD Bioscience | E50-2440 |
| **F4/80** | A450 | Life Technologies /Thermo Fischer scientific | BM8 |
| **IFN-g** | A405 | Life Technologies /Thermo Fischer scientific | XMG1.2 |
| **CD138** | APC | BD Bioscience | 281-2 |
| **CD8a** | Qdot 605 | BD Bioscience | 53-6.7 |
| **Ki67** | A700 | BioLegend | SolA15 |
